# Publisher Correction: A network of chaperones prevents and detects failures in membrane protein lipid bilayer integration

**DOI:** 10.1038/s41467-019-09912-5

**Published:** 2019-04-18

**Authors:** João P. L. Coelho, Matthias Stahl, Nicolas Bloemeke, Kevin Meighen-Berger, Carlos Piedrafita Alvira, Zai-Rong Zhang, Stephan A. Sieber, Matthias J. Feige

**Affiliations:** 1 Center for Integrated Protein Science at the Department of Chemistry, Technical University of Munich, Lichtenbergstr. 4, 85748 Garching, Germany; 2 Interdisciplinary Research Center on Biology and Chemistry, Shanghai Institute of Organic Chemistry, Chinese Academy of Sciences, Shanghai, 201210 China; 3 Institute for Advanced Study, Technical University of Munich, Lichtenbergstr. 2a, 85748 Garching, Germany; 4 SciLifeLab, Department of Oncology-Pathology, Karolinska Institutet, Box 1031, 171 21 Solna Stockholm, Sweden

**Keywords:** Biochemistry, Protein folding, Cell biology

Correction to: *Nature Communications* 10.1038/s41467-019-08632-0, Article published online 8 Feb 2019.

The original version of this Article contained errors in Fig. 1 and Supplementary Fig. 3. In Fig. 1, the labels indicating the Cx32^wt^ constructs in panels d and e were incorrectly shifted with respect to the relevant western blot lanes. In Supplementary Fig. 3, numbers of unique peptides and % sequence coverage were incorrectly reported as being for wt and L90H separately, and should refer to wt and L90H combined. These errors have been corrected in the PDF and HTML versions of the Article.

