# Supplementary material for: A network of chaperones prevents and detects failures in membrane protein lipid bilayer integration
Source: Nat Commun. 2019 Feb 8;10:672. doi: 10.1038/s41467-019-08632-0 (PMC6368539; doi:10.1038/s41467-019-08632-0)
Supplement: Supplementary file 1 — Supplementary Information [file 41467_2019_8632_MOESM1_ESM.pdf]

A network of chaperones prevents and detects failures in membrane protein lipid bilayer integration

João P.L. Coelho[1], Matthias Stahl[1,4], Nicolas Bloemeke[1], Kevin Meighen-Berger[1], Carlos Piedrafita Alvira[1], Zai-Rong Zhang[2], Stephan A. Sieber[1] and Matthias J. Feige[1,3]

[1] Center for Integrated Protein Science at the Department of Chemistry, Technical University of Munich, Lichtenbergstr. 4, 85748 Garching, Germany

[2] Interdisciplinary Research Center on Biology and Chemistry, Shanghai Institute of Organic Chemistry, Chinese Academy of Sciences, Shanghai, 201210, China

[3] Institute for Advanced Study, Technical University of Munich, Lichtenbergstr. 2a, 85748 Garching, Germany

[4] Current address: SciLifeLab, Department of Oncology-Pathology, Karolinska Institutet, Box 1031, 171 21 Solna, Stockholm, Sweden

Correspondence and requests for materials should be addressed to M.J.F. (email: matthias.feige@tum.de)

Supplementary Information

(contains Supplementary Figures 1-6 and Supplementary Table 1 with Legends)

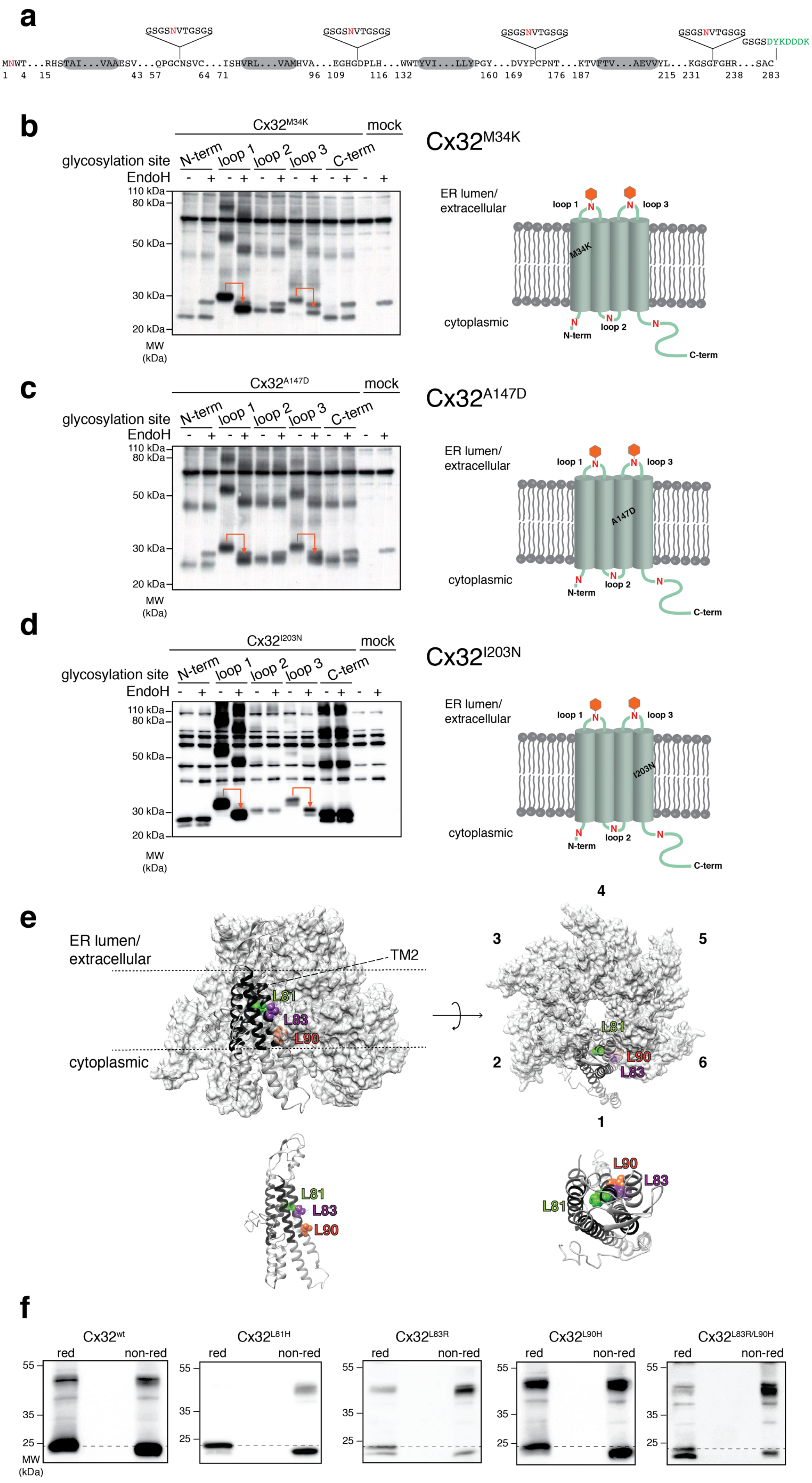
a
GSGSNVTGSGS
GSGSNVTGSGS
GSGSNVTGSGS
GSGSNVTGSGS
GSGSDYKDDDK
MNWT...RHSTAI...VAAESV...QPGCNSVC...ISHVRL...VAMHVA...EGHGDPLH...WWTYVI...LLYPGY...DVYPCPNT...KTVFTV...AEVVYL...KGSGFGHR...SAC
1 4 15 43 57 64 71 96 109 116 132 160 169 176 187 215 231 238 283
b
Cx32M34K
mock
glycosylation site
N-term
loop 1
loop 2
loop 3
C-term
EndoH
110 kDa
80 kDa
50 kDa
30 kDa
20 kDa
MW (kDa)
Cx32M34K
ER lumen/ extracellular
cytoplasmic
loop 1
loop 3
M34K
N-term
loop 2
C-term
c
Cx32A147D
mock
glycosylation site
N-term
loop 1
loop 2
loop 3
C-term
EndoH
110 kDa
80 kDa
50 kDa
30 kDa
20 kDa
MW (kDa)
Cx32A147D
ER lumen/ extracellular
cytoplasmic
loop 1
loop 3
A147D
N-term
loop 2
C-term
d
Cx32I203N
mock
glycosylation site
N-term
loop 1
loop 2
loop 3
C-term
EndoH
110 kDa
80 kDa
50 kDa
30 kDa
20 kDa
MW (kDa)
Cx32I203N
ER lumen/ extracellular
cytoplasmic
loop 1
loop 3
I203N
N-term
loop 2
C-term
e
ER lumen/ extracellular
TM2
L81
L83
L90
cytoplasmic
4
3
5
2
6
1
L81
L90
L83
L81
L83
L90
L90
L83
L81
f
Cx32wt
Cx32L81H
Cx32L83R
Cx32L90H
Cx32L83R/L90H
red
non-red
55
35
25
MW (kDa)

Supplementary Fig. 1. Membrane integration and redox status of Cx32 mutants. **a** Schematic of constructs used. The Cx32 protein sequence is shown with the position of each transmembrane segment highlighted in gray. The N-terminal, naturally occurring glycosylation site, and the ones artificially introduced are shown at their respective positions with a red "N", including linker sequences. The FLAG-tag was placed at the C-terminus of the protein and is shown in green. **b-d** Cx32 mutants with individually introduced glycosylation sites in the indicated regions were transfected into HEK293T cells and treated with or without EndoH as indicated. Samples were run on 10% SDS-PAGE gels and analyzed by immunoblotting with anti-Cx32 antibodies. The three mutants showed the same glycosylation pattern, i.e. topology, as $Cx32^{wt}$ (sites that became glycosylated are indicated with an orange arrow in the blots and an orange hexagon in the schematics on the right). EndoH can produce an unspecific band in immunoblots at ca. 30 kDa, as can be seen in some mock lanes. **e** Side and top view of the modeled Cx32 hexamer and monomer, highlighting the position of the different TM2 mutants studied. While L90 and L83 point towards the interface between two monomer subunits, L81 is predicted to be in the center of the four transmembrane helices forming a monomer. **f** Representative blot of HEK293T cells transfected with the indicated constructs, washed and lysed in the presence of 20 mM NEM (non-red), while reduced (red) samples had β-ME added to the Laemmli buffer. Disulfide bonds seem to be conserved regardless of altered topology between all the variants of the protein, as indicated by the faster mobility observed for the non-reduced samples. Dashed lines were added to highlight migration differences.

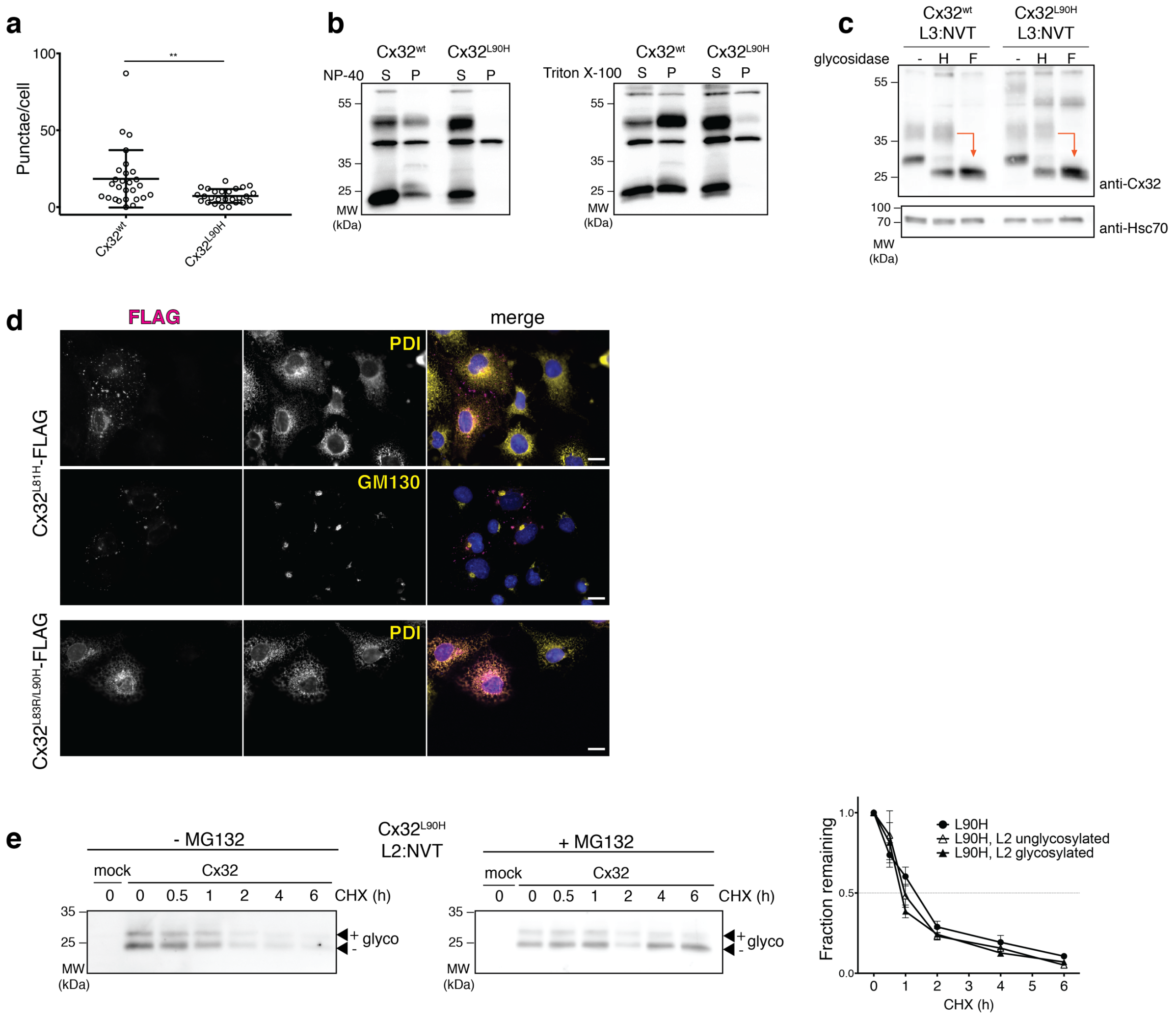

Supplementary Fig. 2. Analysis of gap junction formation, transport and degradation of Cx32 variants**. a** Quantification of punctae in individual cells shows significantly higher numbers for Cx32^{wt}-FLAG when compared to the L90H mutant. Punctae quantification was performed as described in the Methods section. Graphs are presented as mean ± SD, N≥15 cells, ** P value < 0.01, two-tailed Student's t-tests. Note that L90H was generally expressed at lower levels. **b** HEK293T cells transfected with the indicated constructs were lysed in either NP-40 or Triton X-100 lysis buffers, and centrifuged. Supernatants (S) were supplemented with Laemmli+β-ME as described. Pellets (P) were resuspended and dissolved at 95°C for 20 min in 2% SDS containing buffer. Fractions were then diluted in NP-40 or Triton X-100 lysis buffer (P) and supplemented with Laemmli+β-ME. Cx32^{wt} shows signal in the pellet fraction for both detergents, characteristic of the formation of gap junction plaques, which are absent for Cx32^{L90H}. **c** HEK293T cells transfected with the indicated constructs with a

glycosylation site on loop 3 were subjected to digestion by no glycosidase (-), EndoHf (H) or PNGaseF (F). Glycosylation in loop 3 was observed to be further modified in the Golgi complex in $Cx32^{wt}$ and $Cx32^{L90H}$, as seen by the EndoHf-resistant smear detectable above the 35 kDa marker. Hsc70 was used as a loading control. Orange arrows show the downwards shift of complex modified glycans upon PNGaseF treatment. **d** COS-7 cells were transfected with either $Cx32^{L81H}$ or $Cx32^{L83R/L90H}$, immunostained for FLAG-tagged Cx32 (magenta), PDI (yellow) as an ER marker, or GM130 (yellow) as a Golgi maker. Nuclei were stained with DAPI (blue). Anti-FLAG immunofluorescence data are depicted as maximum intensity projections from deconvoluted z-stacks for $Cx32^{L81H}$, while FLAG immunofluorescence signals from $Cx32^{L83R/L90H}$, as well as from PDI, GM130 and nuclei are shown as a central cell plane from the non-deconvoluted images. Pictures are representative of cells from at least three different biological replicates. Scale bars correspond to 20 μm. **e** HEK293T cells transfected with the indicated constructs were incubated with either 50 μg/ml CHX, or 10 μM MG132 for an initial period of 3 h, followed by CHX and MG132 co-incubation for the indicated times, before cell lysis and protein separation on a 10% SDS-PAGE gel. Arrowheads indicate monomer and glycosylated monomer bands quantified to determine Cx32 turnover. The graph on the right shows degradation curves corresponding to aforementioned species, normalized to the first time point. “L90H” data are taken from Figure 2. Introduction of the artificial glycosylation reporter site and glycosylation itself thus do not have an effect on $Cx32^{L90H}$ degradation. Data points correspond to mean ± SEM, N≥4.

a

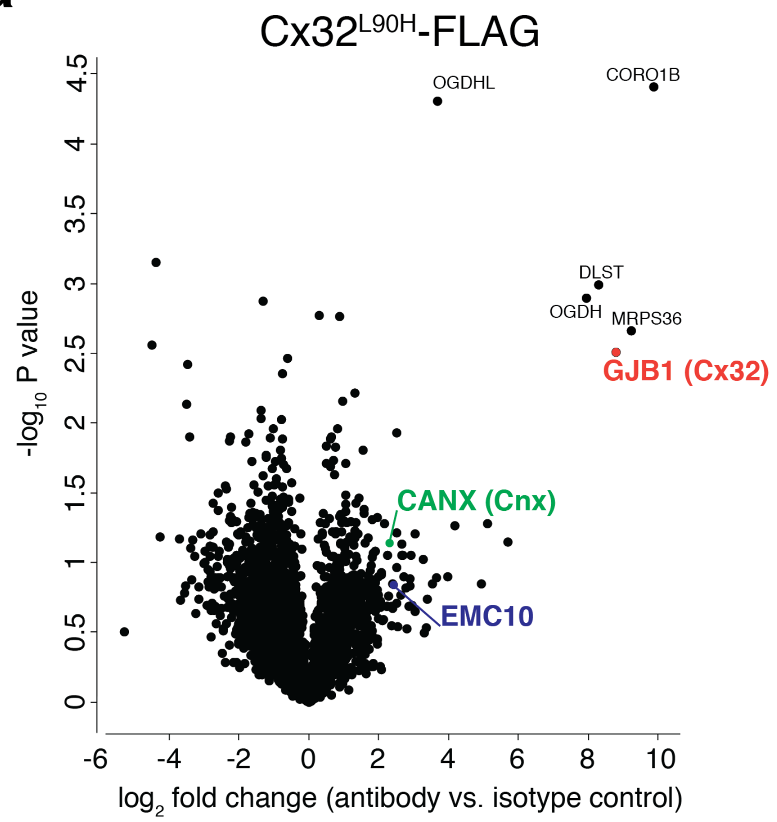

b

	log_2 LFQ intensity anti-FLAG (replicates)		log_2 LFQ intensity isotype control (replicates)		log_2 fold change		$-log_{10}$ P value		number of peptides (unique)	sequence coverage (%)
	wt	L90H	wt	L90H	wt	L90H	wt	L90H	combined	combined
Cx32	29.3178<br>29.1056<br>29.8344	27.2016<br>28.2512<br>28.7193	16.9197<br>19.7542<br>19.5515	17.5926<br>21.8387<br>*18.3359*	10.6774	8.80164	3.46852	2.50402	13 (13)	42.8
Cnx	27.2947<br>26.2045<br>27.5619	25.306<br>24.0994<br>26.6546	*20.7605*<br>22.3343<br>23.1601	24.2348<br>22.2214<br>22.6476	4.93536	2.31874	2.42136	1.13697	15 (15)	27.9
EMC10	22.3115<br>22.485<br>22.1873	*19.3048*<br>23.6643<br>22.1022	*19.0374*<br>*18.7427*<br>*17.9319*	*18.7573*<br>*20.0582*<br>*18.9963*	3.75727	2.4198	3.41077	0.84034	2 (2)	7.8

Supplementary Fig. 3. Interaction partners of Cx32 identified by mass spectrometry. **a** Volcano plot, as shown in Fig. 3, of proteins identified by mass spectrometry after $Cx32^{L90H}$-FLAG immunoprecipitation in 1% digitonin. Proteins whose interaction was studied further are highlighted. **b** Quantitative parameters of the mass spectrometry measurements for Cx32 and two of the interaction partners studied, shown for both $Cx32^{wt}$ and $Cx32^{L90H}$ (imputed values are shown in italic).

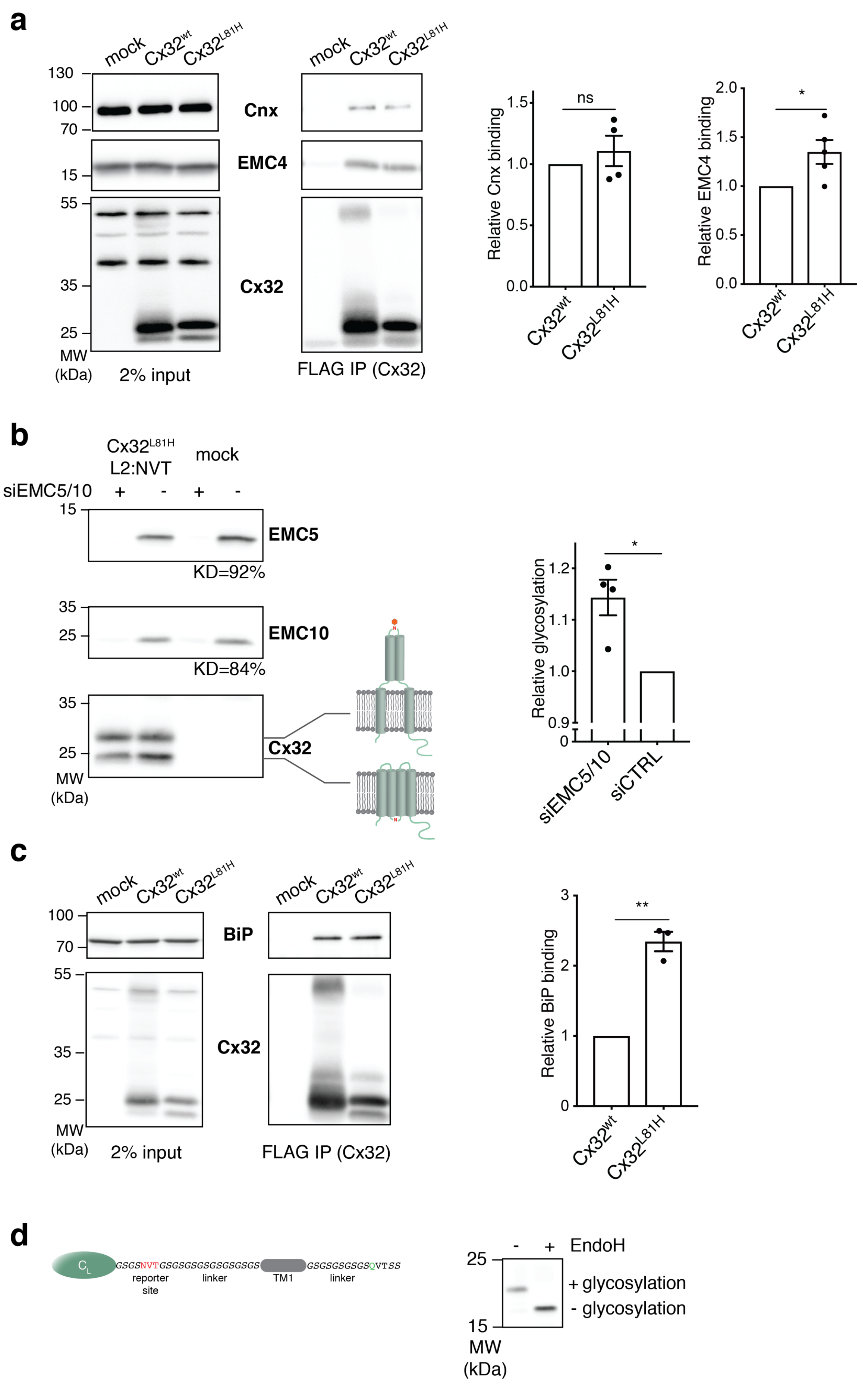
a
mock
Cx32wt
Cx32L81H
130
100
70
15
55
35
25
MW
(kDa)
Cnx
EMC4
Cx32
2% input
FLAG IP (Cx32)
Relative Cnx binding
ns
Relative EMC4 binding
*
b
Cx32L81H
L2:NVT
siEMC5/10
+
-
EMC5
KD=92%
EMC10
KD=84%
Relative glycosylation
siEMC5/10
siCTRL
c
BiP
Relative BiP binding
**
d
CL
GSGSNVTGSGSGSGSGSGSGS
GSGSGSGSGSQVTSS
reporter
site
linker
TM1
EndoH
+ glycosylation
- glycosylation

Supplementary Fig. 4. $Cx32^{L81H}$ shows similar interactions with ER chaperones like $Cx32^{L90H}$. **a** Representative blots from FLAG immunoprecipitation experiments in HEK293T cells transfected with the indicated constructs. $Cx32^{L81H}$ interacts with endogenous Cnx and EMC4 in a comparable fashion to $Cx32^{L90H}$ (mean ± SEM, N=4-5, * P value < 0.05, two-tailed Student's t-tests). Quantifications were performed as described in the Methods section. **b** Transient knockdown of EMC5/10 by siRNA (average knockdown (KD) efficiencies are shown below the blots) increases glycosylation of a reporter site in loop 2 for $Cx32^{L81H}$. Monomeric species +/- glycosylation as a reporter for the topology (indicated on the right) are shown on the blot. Changes in glycosylation, quantified as described in the Methods section, are shown on the right (mean ± SEM, N=4, * P value < 0.05, two-tailed Student's t-tests). **c** Same as in **a** for co-transfected hamster BiP (mean ± SEM, N=3, ** P value < 0.01, two-tailed Student's t-tests). **d** The C_L-TM1 construct adopts a type I topology in the ER membrane. In order to assess if inversion of the TM1 helix could occur, since C_L-TM1 construct design involved inverting the sequence of the respective helix, a glycosylation site (NVT) was placed N-terminally of the TM segment, and the original (Fig. 4) glycosylation site was mutated (Q shown in green). Transfection of this construct into HEK293T cells, followed by lysis and EndoH digestion, revealed quantitative glycosylation of the new site, corroborating a type I topology.

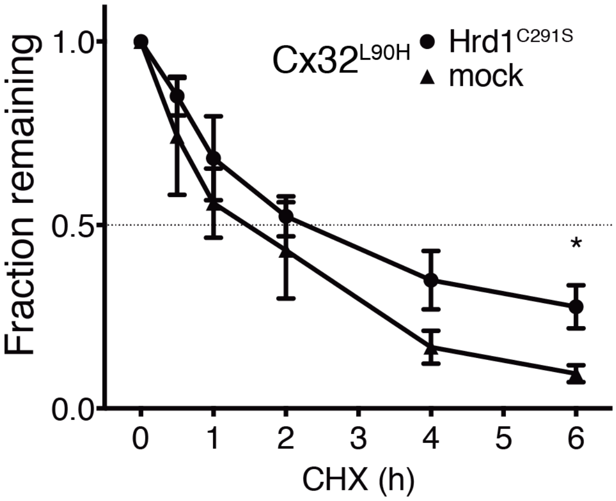

Supplementary Fig. 5. HEK293T cells were co-transfected with Cx32^{L90H} and Hrd1^{C291S}. Cells were incubated with CHX and lysed at the indicated time points. Non-functional Hrd1^{C291S} slows down Cx32 degradation, although to a lesser extent than non-functional gp78 (see Figure 4; mean ± SEM, N=5, * P value < 0.05, two-tailed Student's t-tests).

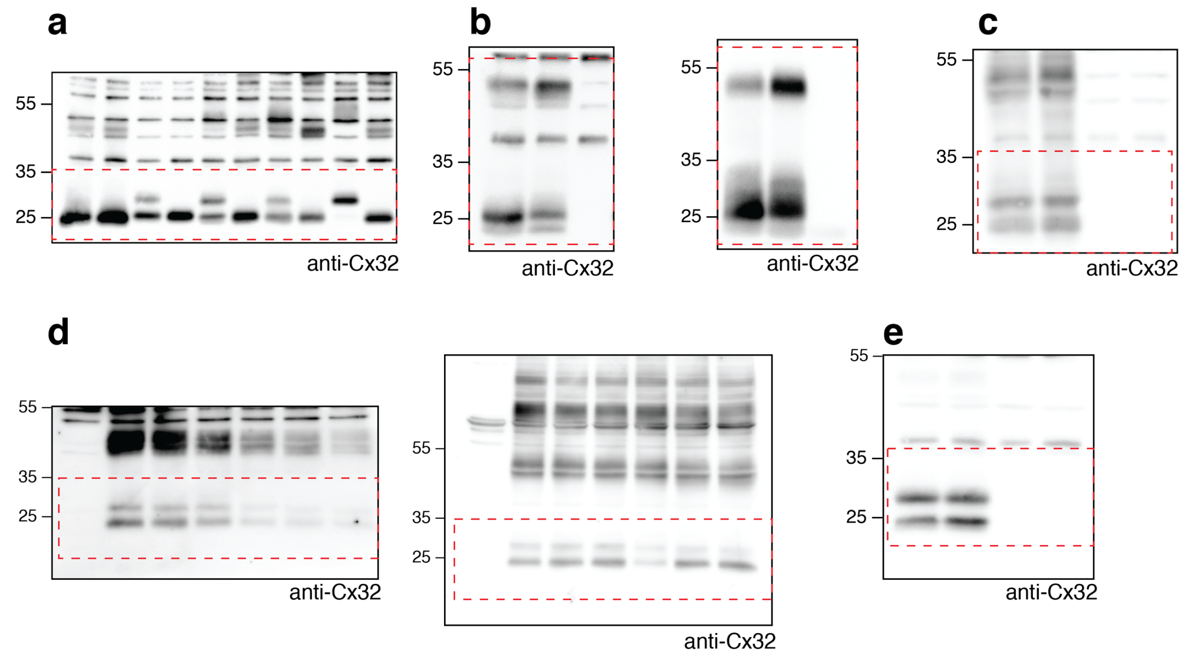

Supplementary Fig. 6. Original uncropped blots. Blots where significant crops were performed are presented, with a red box indicating the part of the blots shown in the respective figure. Blots shown belong to **a** Fig. 1f, **b** Fig. 3b, **c** Fig. 3c, **d** Supplementary Fig. 2e, and **e** Supplementary Fig. 4b. Molecular weight markers (kDa) are indicated at the left side of the blots, primary antibodies used for detection are written below the immunoblots.

Cx32 mutagenic primers	
M34K (for)<br>M34K (rev)	CTTCATCTTCAGAATCAAGGTGCTGGTGGTGGCTG<br>CAGCCACCACCAGCACCTTGATTCTGAAGATGAAG
L81H (for)<br>L81H (rev)	CTGTGGTCCCTGCAGCACATCCTAGTTTCCAC<br>GTGGAAACTAGGATGTGCTGCAGGGACCACAG
L83R (for)<br>L83R (rev)	CTGCAGCTCATCCGAGTTTCCACCCCAG<br>CTGGGGTGGAAACTCGGATGAGCTGCAG
L90H (for)<br>L90H (rev)	GTTTCCACCCCAGCTCTCCACGTGGCCATGCACGTGG<br>CCACGTGCATGGCCACGTGGAGAGCTGGGGTGGAAAC
A147D (for)<br>A147D (rev)	CGGCTGTTGTTTGAGGACGTCTTCATGTATGTC<br>GACATACATGAAGACGTCCTCAAACAACAGCCG
I203N (for)<br>I203N (rev)	CTGGCATCTGCATCAACCTCAATGTGGCCGAG<br>CTCGGCCACATTGAGGTTGATGCAGATGCCAG
Loop1:NVT (for)<br><br>Loop1:NVT (rev)	CAACACACTCCAGCCTGGCTGCGGCAGCGGATCCAACGTGACCGGC<br>AGCGGATCCAACAGCGTTTGCTATGACC<br>GGTCATAGCAAACGCTGTTGGATCCGCTGCCGGTCACGTTGGATCC<br>GCTGCCGCAGCCAGGCTGGAGTGTGTTG
Loop2:NVT (for)<br><br>Loop2:NVT (rev)	CGGCTTGAGGGCCATGGGGGCAGCGGATCCAACGTGACCGGCAGCGGCTC<br>CGACCCCCTACACCTGGAGG<br>CCTCCAGGTGTAGGGGGTCGGAGCCGCTGCCGGTCACGTTGGATCCGCTG<br>CCCCCATGGCCCTCAAGCCG
Loop3:NVT (for)<br><br>Loop3:NVT (rev)	GTCAAGTGCGACGTCTACCCCGGCAGCGGATCCAACGTGACCGGCAGCGG<br>ATCCTGCCCCAACACAGTGGACTG<br>CAGTCCACTGTGTTGGGGCAGGATCCGCTGCCGGTCACGTTGGATCCGCT<br>GCCGGGGTAGACGTCGCACTTGAC
Cterm:NVT (for)<br><br>Cterm:NVT (rev)	CACCTTCCCGCAAGGGCTCGGGCGGCAGCGGATCCAACGTGACCGGCAGC<br>GGATCCTTCGGCCACCGCCTCTCACC<br>GGTGAGAGGCGGTGGCCGAAGGATCCGCTGCCGGTCACGTTGGATCCGCT<br>GCCGCCCGAGCCCTTGCGGGAAGGTG

Other mutagenic primers	
C_L TM1-QVT (for)<br>C_L TM1-QVT (rev)	TGGCTCCGGCTCTGGCAGCCAAGTGACCAGCTCTTGATGAGG<br>CCTCATCAAGAGCTGGTCACTTGGCTGCCAGAGCCGGAGCCA
C_L NVT-TM1 (for)<br><br>C_L NVT-TM1 (rev)	CCTGAGCAGAGCCGATTCTAGAGGCTCTGGAAGCAATGTGACCGGAAGCG<br>GATCTGGCTCTGGCAGCGGCTCTGGAAGCGGA<br>TCCGCTTCCAGAGCCGCTGCCAGAGCCAGATCCGCTTCCGGTCACATTGC<br>TTCCAGAGCCTCTAGAATCGGCTCTGCTCAGG
C_L $TM2^{L90H}$ (for)<br>C_L $TM2^{L90H}$ (rev)	GTGTCTACACCAGCTCTGCACGTGGCCATGCACGTTGG<br>CCAACGTGCATGGCCACGTGCAGAGCTGGTGTAGACAC
$gp78^{C341S}$ (for)<br>$gp78^{C341S}$ (rev)	GCTGTCAACAATGACGACTCTGCCATCTGTTGGGACT<br>AGTCCCAACAGATGGCAGAGTCGTCATTGTTGACAGC
$gp78^{C378S}$ (for)<br>$gp78^{C378S}$ (rev)	ACACCTCCTGTCCAACATCCAGAATGTCTCTTAATATTGCC<br>GGCAATATTAAGAGACATTCTGGATGTTGGACAGGAGGTGT

Cloning primers	
Cx32 XhoI (for)	TATACTCGAGGCCACCATGAACTGGACAGGTTTGTACACCTTG
Cx32 BamHI (rev)	AAAAGGATCCTTATCAGCAGGCCGAGCAGCGGTCGCTCTTTTC
Cx32 FLAG insertion (rev)	GGGGCTGGGCTGGCTGAAAAGAGCGACCGCTGCTCGGCCTGCGGCAGCGG<br>AAGCGACTACAAAGACGATGACGACAAGTGATAAGGATCCAGACATGATA<br>AGATACATTGATGAGTTTGG

Supplementary Table 1. Primers used in this study. All primers are shown in a 5' to 3' orientation.
