# Supplementary material for: A network of chaperones prevents and detects failures in membrane protein lipid bilayer integration
Source: Nat Commun. 2019 Feb 8;10:672. doi: 10.1038/s41467-019-08632-0 (PMC6368539; doi:10.1038/s41467-019-08632-0)
Supplement: Supplementary file 3 — Reporting Summary [file 41467_2019_8632_MOESM3_ESM.pdf]

nature research

Corresponding author(s): Feige, Matthias J

Last updated by author(s): Jan 20, 2019

Reporting Summary

Nature Research wishes to improve the reproducibility of the work that we publish. This form provides structure for consistency and transparency in reporting. For further information on Nature Research policies, see Authors & Referees and the Editorial Policy Checklist.

Statistics

For all statistical analyses, confirm that the following items are present in the figure legend, table legend, main text, or Methods section.

n/a	Confirmed	
☐	☒	The exact sample size (*n*) for each experimental group/condition, given as a discrete number and unit of measurement
☐	☒	A statement on whether measurements were taken from distinct samples or whether the same sample was measured repeatedly
☐	☒	The statistical test(s) used AND whether they are one- or two-sided<br>*Only common tests should be described solely by name; describe more complex techniques in the Methods section.*
☒	☐	A description of all covariates tested
☐	☒	A description of any assumptions or corrections, such as tests of normality and adjustment for multiple comparisons
☐	☒	A full description of the statistical parameters including central tendency (e.g. means) or other basic estimates (e.g. regression coefficient) AND variation (e.g. standard deviation) or associated estimates of uncertainty (e.g. confidence intervals)
☐	☒	For null hypothesis testing, the test statistic (e.g. *F*, *t*, *r*) with confidence intervals, effect sizes, degrees of freedom and *P* value noted<br>*Give P values as exact values whenever suitable.*
☒	☐	For Bayesian analysis, information on the choice of priors and Markov chain Monte Carlo settings
☒	☐	For hierarchical and complex designs, identification of the appropriate level for tests and full reporting of outcomes
☒	☐	Estimates of effect sizes (e.g. Cohen's *d*, Pearson's *r*), indicating how they were calculated

Our web collection on statistics for biologists contains articles on many of the points above.

Software and code

Policy information about availability of computer code

Data collection	For data collection, always standard software of the respective equipment was used. Whenever deviating from this, software is explicitly mentioned in the manuscript.
Data analysis	All software used to analyze data is mentioned explicitly, including version numbers, in the manuscript.

For manuscripts utilizing custom algorithms or software that are central to the research but not yet described in published literature, software must be made available to editors/reviewers. We strongly encourage code deposition in a community repository (e.g. GitHub). See the Nature Research guidelines for submitting code & software for further information.

Data

Policy information about availability of data

All manuscripts must include a data availability statement. This statement should provide the following information, where applicable:

- Accession codes, unique identifiers, or web links for publicly available datasets
- A list of figures that have associated raw data
- A description of any restrictions on data availability

Data supporting the findings of this manuscript are available from the corresponding author upon reasonable request. A reporting summary for this Article is available as a Supplementary Information file. Mass spectrometry proteomics data from this study have been deposited to the ProteomeXchange Consortium (http://proteomecentral.proteomexchange.org) with the dataset identifier PXD009189 (login credentials upon request).

Field-specific reporting

Please select the one below that is the best fit for your research. If you are not sure, read the appropriate sections before making your selection.

☒ Life sciences ☐ Behavioural & social sciences ☐ Ecological, evolutionary & environmental sciences

For a reference copy of the document with all sections, see nature.com/documents/nr-reporting-summary-flat.pdf

Life sciences study design

All studies must disclose on these points even when the disclosure is negative.

Sample size	Sample sizes were determined according to each individual experiment.
Data exclusions	No data was excluded for any experiment.
Replication	All findings were completely reproducible in independent biological replicates.
Randomization	No randomization was performed.
Blinding	No blinding was performed.

Reporting for specific materials, systems and methods

We require information from authors about some types of materials, experimental systems and methods used in many studies. Here, indicate whether each material, system or method listed is relevant to your study. If you are not sure if a list item applies to your research, read the appropriate section before selecting a response.

Materials & experimental systems

n/a	Involved in the study	
☐	☒	Antibodies
☐	☒	Eukaryotic cell lines
☒	☐	Palaeontology
☒	☐	Animals and other organisms
☒	☐	Human research participants
☒	☐	Clinical data

Methods

n/a	Involved in the study	
☒	☐	ChIP-seq
☒	☐	Flow cytometry
☒	☐	MRI-based neuroimaging

Antibodies

Antibodies used	All antibodies used are described in detail in the Methods section, either giving the details on the supplier & product number or the contact of the research group who provided the antibody.
Validation	All commercial antibodies were validated by the supplier; additionally, most were independently valiadated by us, using knock-down experiments of the protein of interest or mock samples as negative controls. Antibodies derived from other research groups (BiP, Hendershot lab) have been extensively validated in the literature.

Eukaryotic cell lines

Policy information about cell lines

Cell line source(s)	ECACC (via Sigma Aldrich)
Authentication	Authentication by the supplier.
Mycoplasma contamination	All cell lines were regularly tested for mycoplasma and always found to be negative.
Commonly misidentified lines (See ICLAC register)	None.
